# Supplementary material for: AUNIP/C1orf135 directs DNA double-strand breaks towards the homologous recombination repair pathway
Source: Nat Commun. 2017 Oct 17;8:985. doi: 10.1038/s41467-017-01151-w (PMC5645412; doi:10.1038/s41467-017-01151-w)
Supplement: Supplementary file 1 — Supplementary Information [file 41467_2017_1151_MOESM1_ESM.pdf]

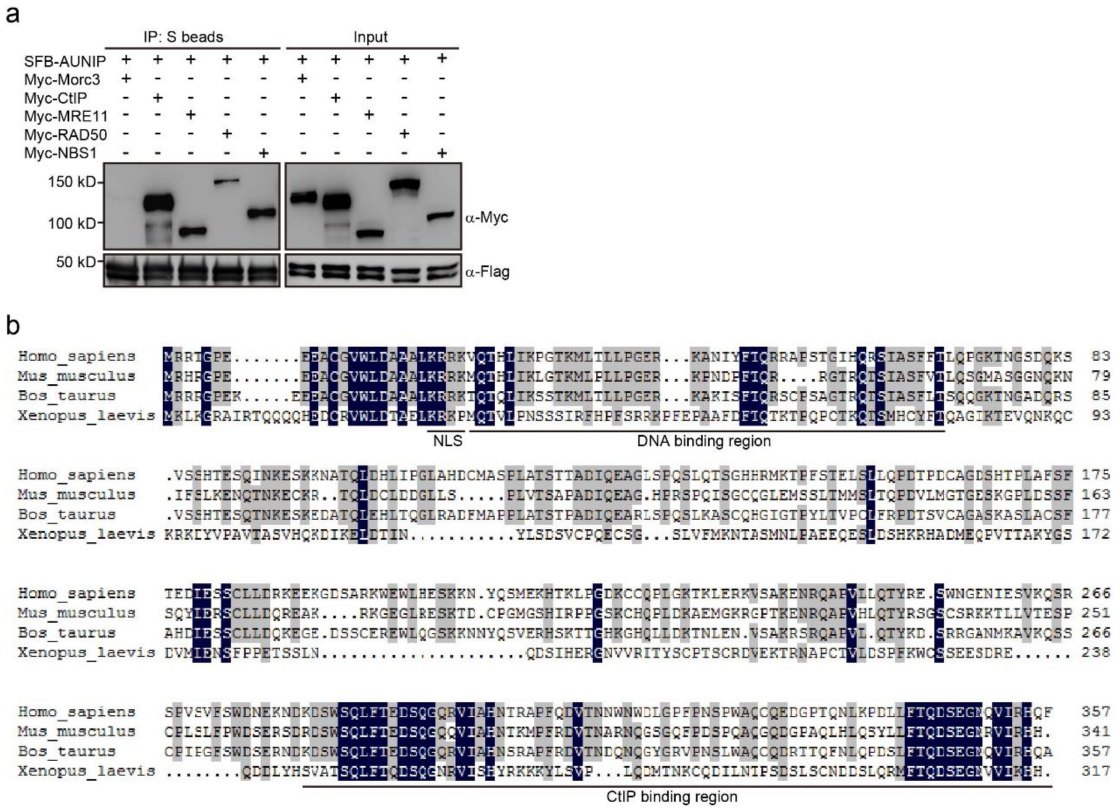

Supplementary Figure 1. AUNIP interacts with CtIP and the MRN complex. (**a**) HEK293T cells transfected with indicated plasmids were lysed with NETN buffer. Cell lysates were then incubated with S protein beads and western blot analysis was performed with indicated antibodies. (**b**) Amino acid sequence alignment of AUNIP from various species.

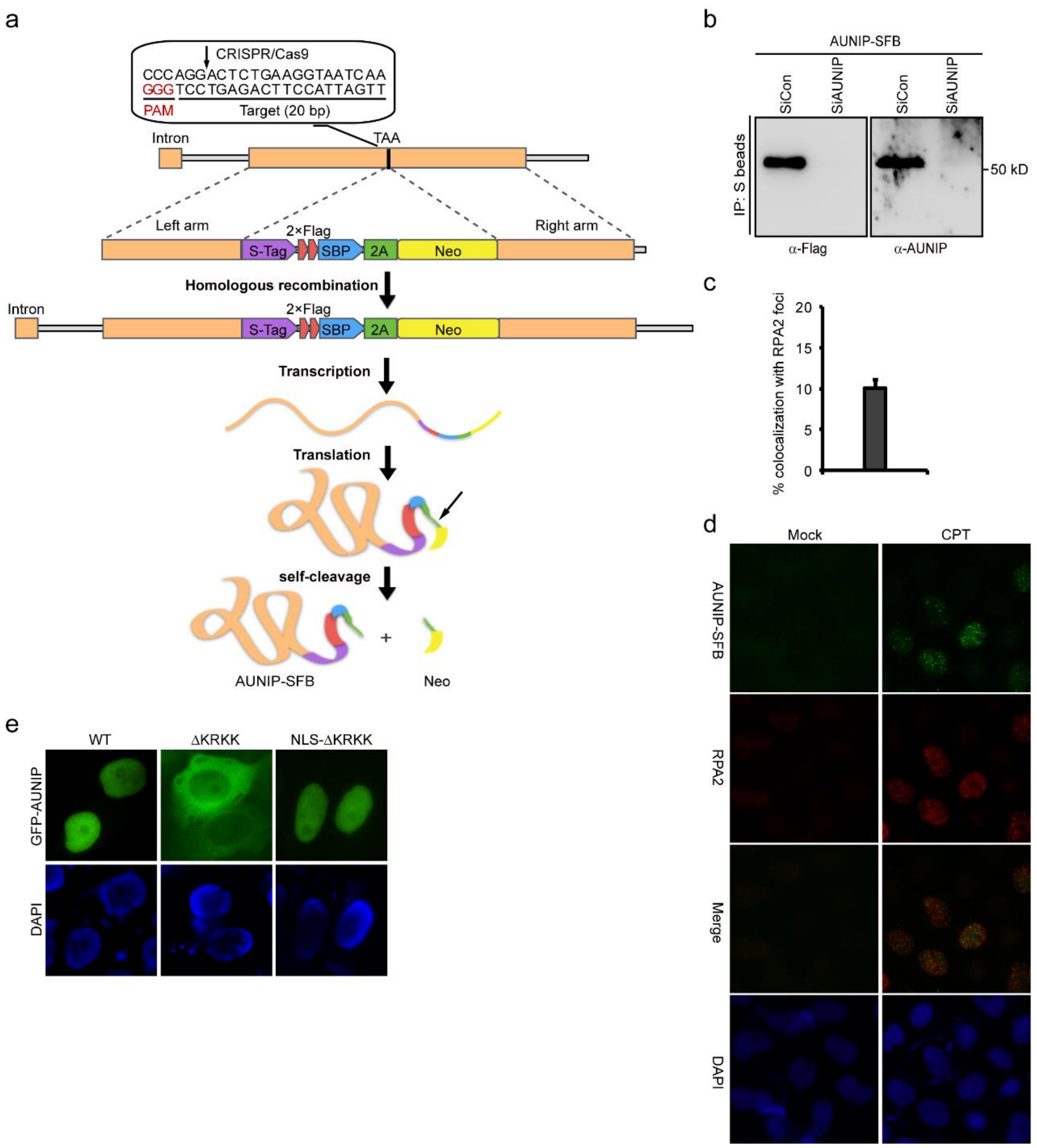

Supplementary Figure 2. AUNIP accumulates at sites of DNA damage. (**a**) Strategy for generation of a AUNIP-SFB knock-in HeLa cell line. (**b**) AUNIP-SFB knock-in HeLa cells were transfected with indicated siRNAs and 48 hr later cell lysates were immunoprecipitated with S protein beads and probed with anti-Flag or anti-AUNIP antibody. (**c**) AUNIP foci partially colocalized with RPA2 foci. Data are presented as mean ± SEM for three independent experiments. (**d**) AUNIP foci are found exclusively in RPA2 foci-positive cells. AUNIP-SFB knock-in HeLa cells were treated with 1 μM CPT for 6 hr before being processed for AUNIP-SFB and RPA2 immunofluorescence. (**e**) Amino acids 21-24 (KRKK) of AUNIP constitute a nuclear localization sequence (NLS) and were required for its nuclear localization. Cells were transfected with GFP-tagged wild-type AUNIP, the ΔKRKK mutant (lacking amino acids 21-24), or the NLS-ΔKRKK mutant (the KRKK sequence were added back to the N-terminal end of the ΔKRKK construct). At 24 hr after transfection, the cells were processed for immunofluorescence.

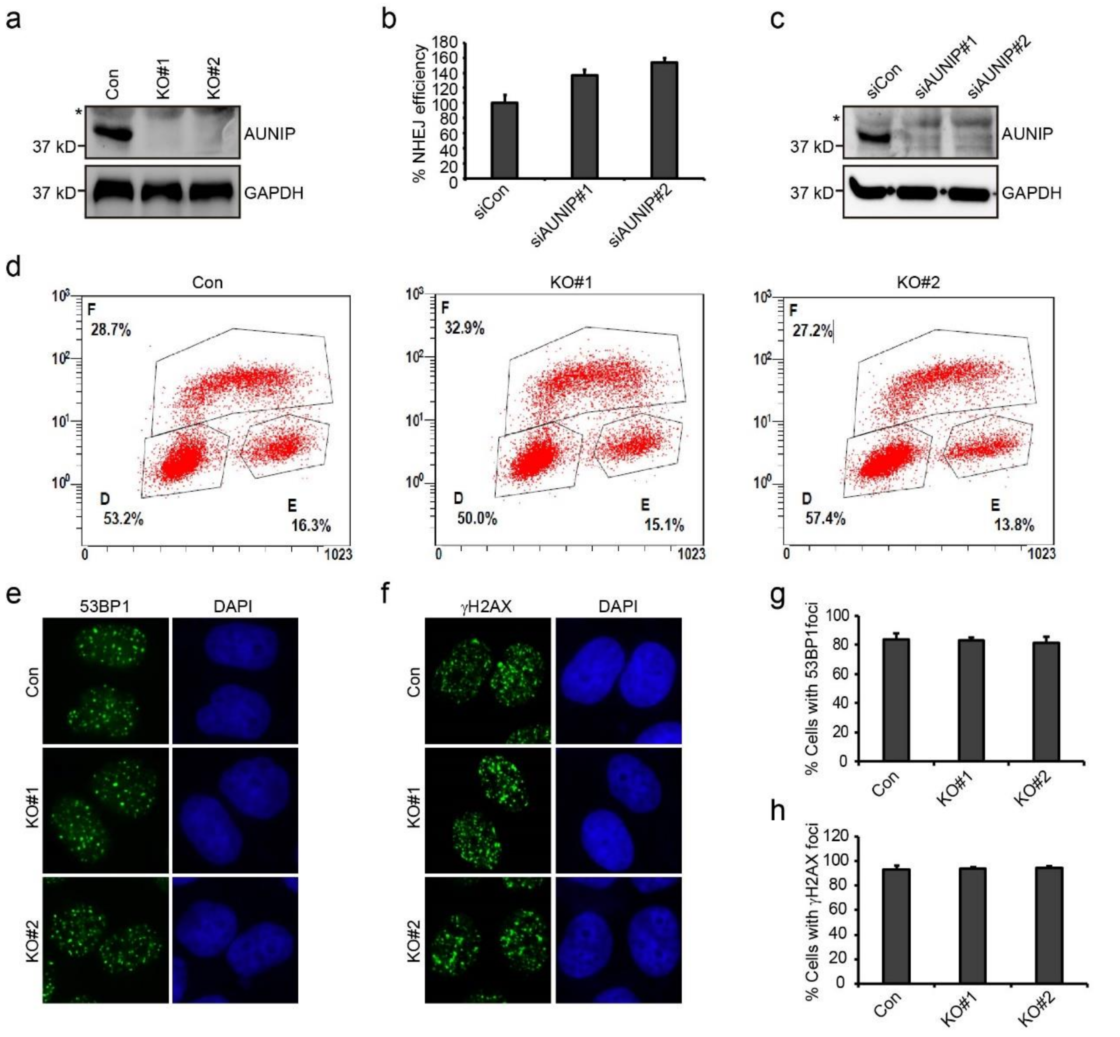

Supplementary Figure 3. AUNIP inhibits NHEJ. (**a**) Knockout efficiency of AUNIP was confirmed by western blotting. Asterisks indicate nonspecific bands. (**b-c**) AUNIP depletion increases NHEJ. U2OS EJ5-GFP cells transfected with indicated siRNAs were electroporated with an I-SceI expression plasmid for 48 hr before they were assayed for HR efficiency. Data are presented as mean ± SEM for three independent experiments (**b**). Knockdown efficiency was confirmed by immunoblotting (**c**). (**d**) AUNIP knockout does not affect cell cycle phase distribution. F refers to S phase, D refers to G1 phase, and E refers to G2/M phase. (**e-h**) AUNIP knockout has no effect on 53BP1 and γH2AX foci formation. Wild-type and AUNIP-deficient HeLa cells were treated with 10Gy X-rays and allowed to recover for 1 hr before being processed for 53BP1 (**e**) or γH2AX (**f**) immunofluorescence. Data are presented as mean ± SEM for three independent experiments (**g** and **h**).

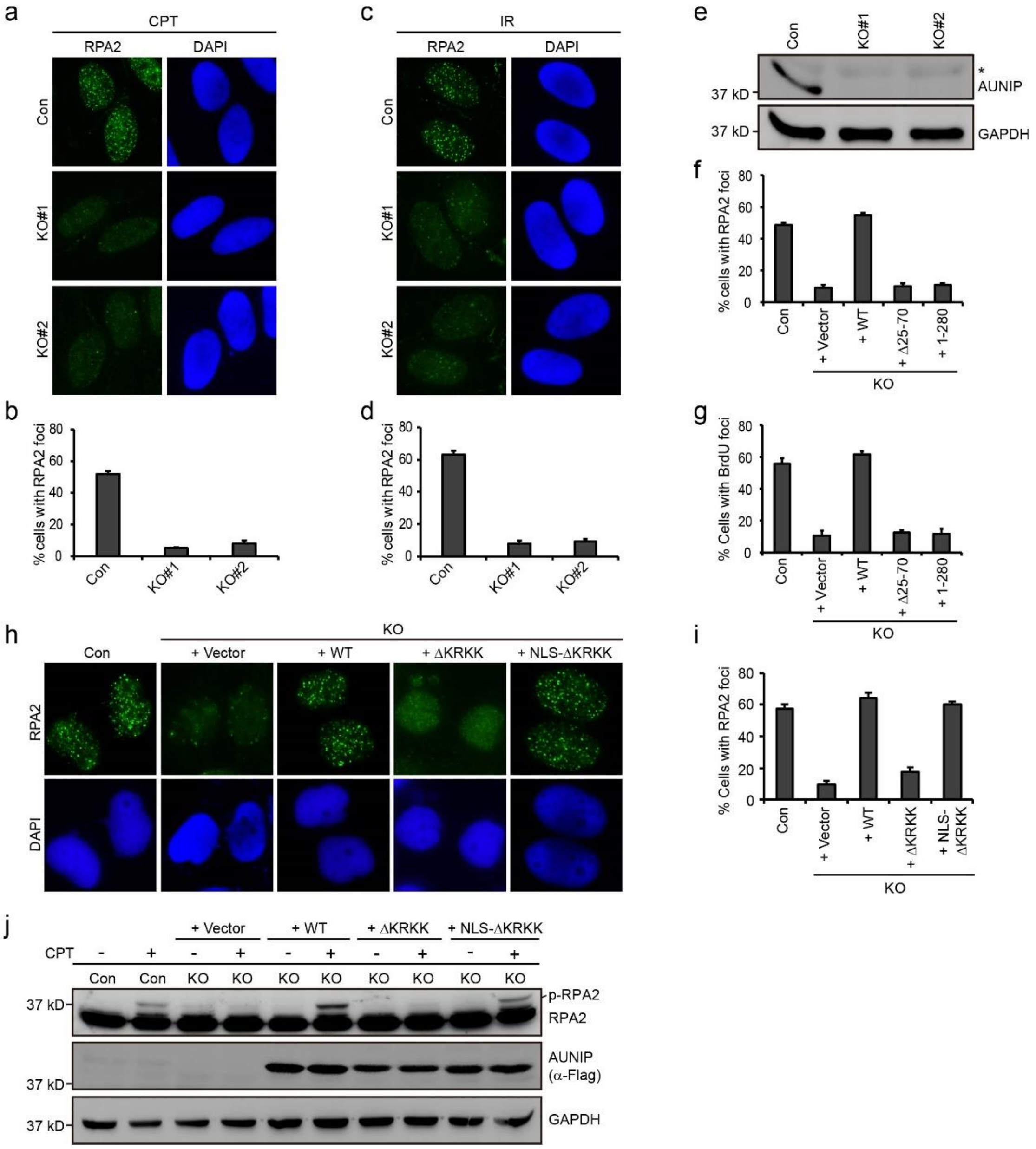

Supplementary Figure 4. AUNIP promotes DNA end resection in U2OS cells. (**a-d**) Cells were treated with CPT or X-rays for 1 hr before being processed for RPA2 immunofluorescence (**a** and **c**). Data are presented as mean ± SEM for three independent experiments (**b** and **d**). (**e**) Knockout efficiency of AUNIP was confirmed by western blotting. Asterisks indicate nonspecific bands. (**f-g**) The mutants lacking the ability to localize to DNA damage sites or to bind to CtIP of AUNIP failed to rescue RPA2/BrdU foci formation in AUNIP-deficient cells. Cells were treated with CPT for 1 hr before being processed for RPA2/BrdU immunofluorescence. Data are presented as mean ± SEM for three independent experiments. (**h-j**) The NLS of AUNIP is required for RPA2 foci formation and phosphorylation. Cells were treated with CPT for 1 hr and then subjected to immunostaining (**h**) or Western blotting (**j**) using indicated antibodies. Data are presented as mean ± SEM for three independent experiments (**i**).

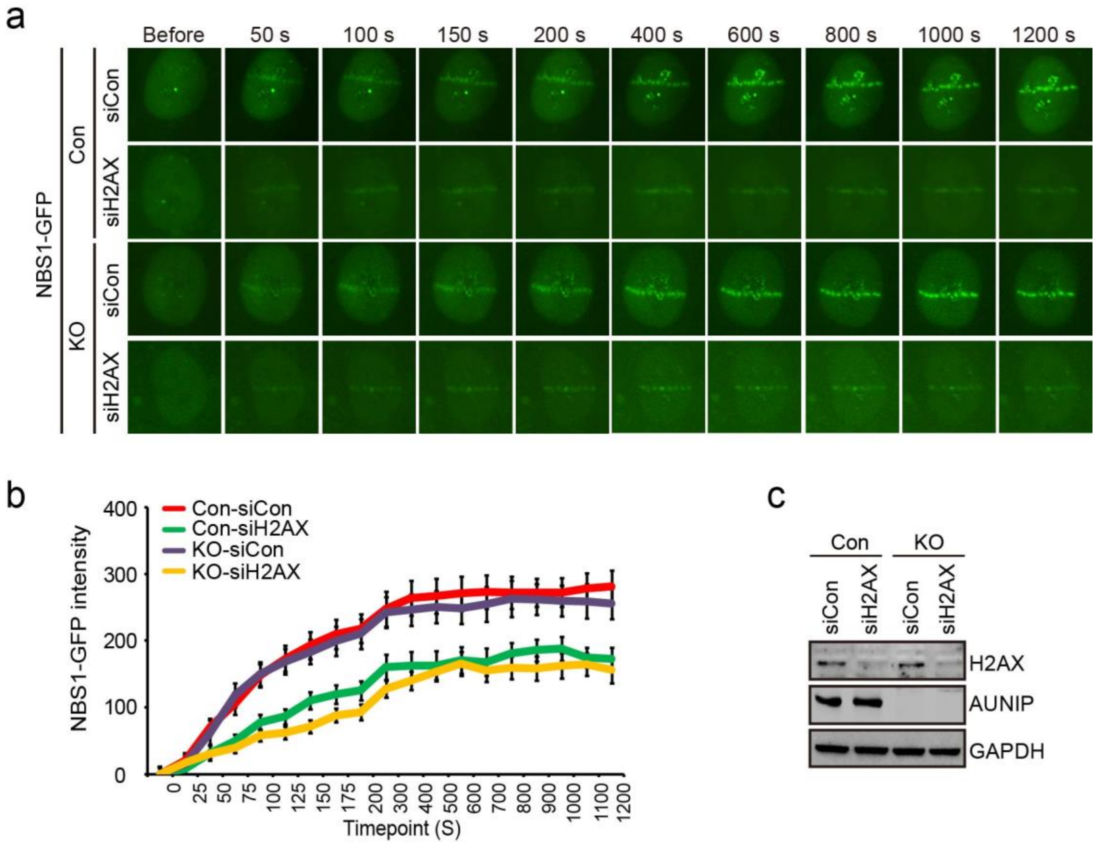

Supplementary Figure 5. AUNIP silencing did not significantly affect NBS1 recruitment to laser-induced DNA damage sites. (**a**) Wild-type or AUNIP-deficient cells infected with a lentiviral vector expressing NBS1-GFP were transfected with indicated siRNAs. After 48 hr, cells were laser micro-irradiated and monitored by living cell imaging. Representative images are shown. (**b**) The intensity of fluorescence at the site of damage was quantified. Data are analyzed from at least 20 cells in each experiment and are presented as mean ± SEM. (**c**) Knockdown efficiency of H2AX was confirmed by immunoblotting.

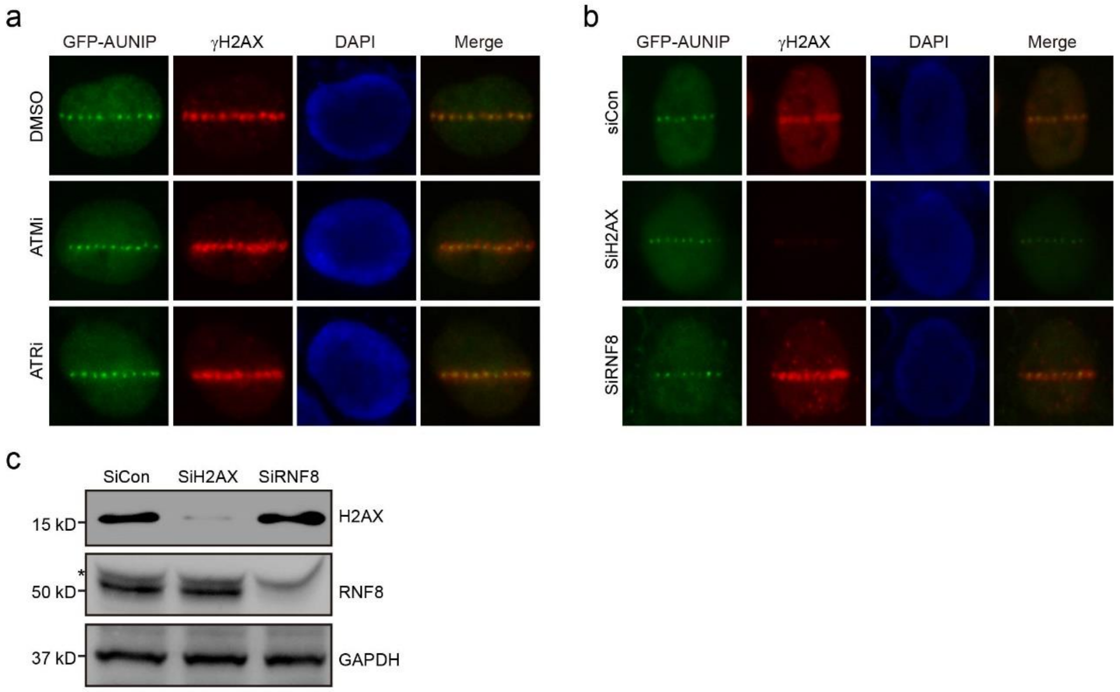

Supplementary Figure 6. AUNIP damage recruitment is independent on the ATM and ATR kinase pathways. (**a**) U2OS cells stably expressing GFP-AUNIP were either mock treated or treated with ATM/ATR inhibitor (KU-60019 and VE-821, respectively) (10 μM) for 1 hr. Cell were then laser micro-irradiated and stained with anti-γH2AX antibody. (**b**) Neither H2AX nor RNF8 is required for AUNIP damage recruitment. U2OS cells stably expressing GFP-AUNIP were transfected twice with control siRNA, or siRNAs specific for H2AX/RNF8. 48 hrs later, cells were laser micro-irradiated and stained with anti-γH2AX antibody. (**c**) Knockdown efficiency of H2AX/RNF8 was confirmed by western blotting. Asterisks indicate nonspecific bands.

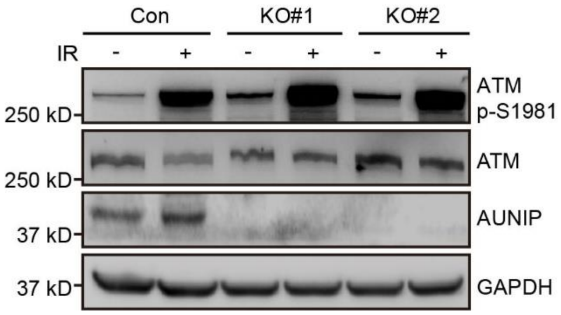

Supplementary Figure 7. AUNIP is not required for ATM activation. Phosphorylation of ATM upon AUNIP silencing was analyzed by western blotting of lysates prepared from indicated cells at 1 hr after cells exposure to 10 Gy X-rays.

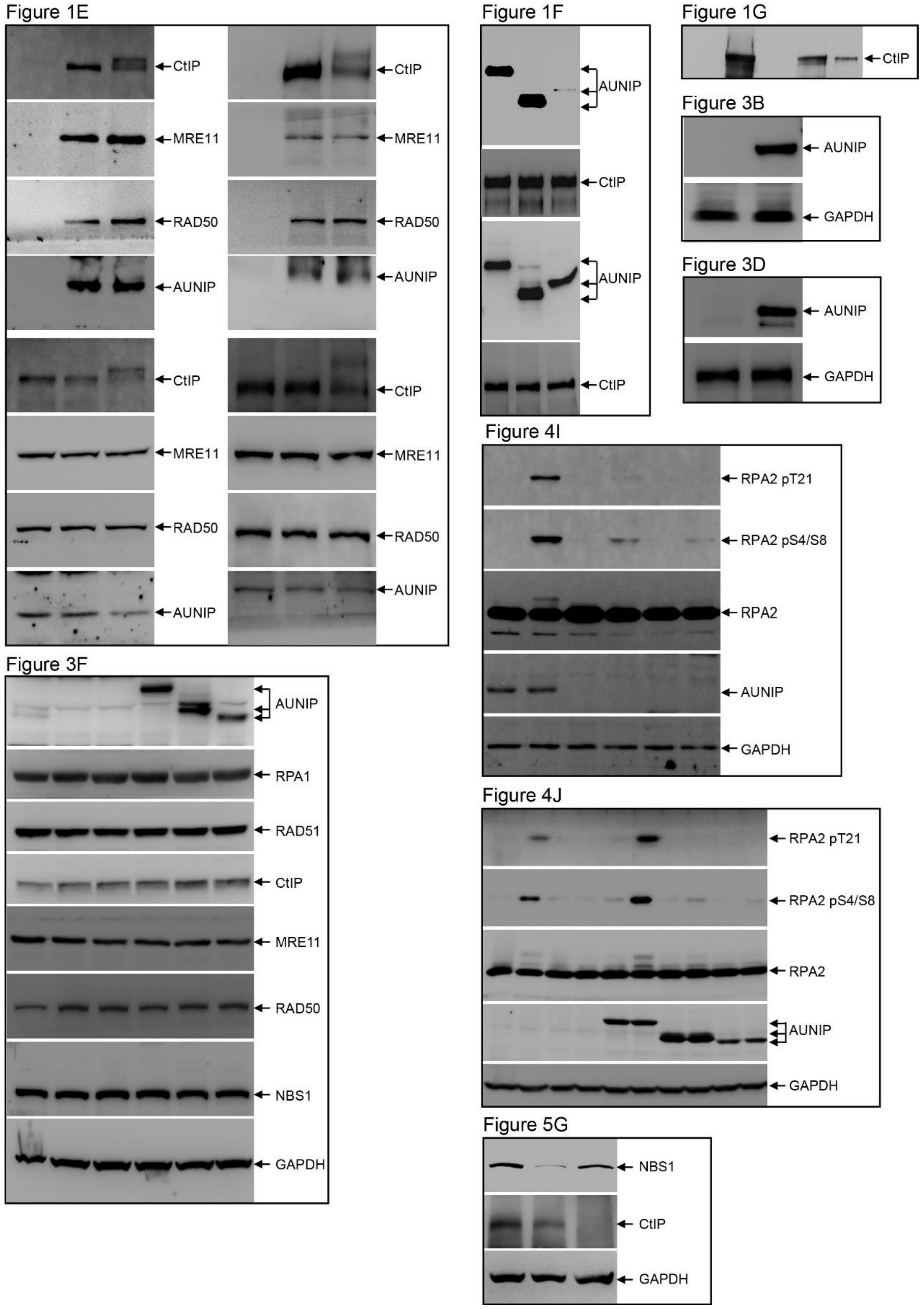

Supplementary Figure 8. Uncropped immunoblots.
