# Supplementary material for: AUNIP/C1orf135 directs DNA double-strand breaks towards the homologous recombination repair pathway
Source: Nat Commun. 2017 Oct 17;8:985. doi: 10.1038/s41467-017-01151-w (PMC5645412; doi:10.1038/s41467-017-01151-w)
Supplement: Supplementary file 3 — Supplementary Files [file 41467_2017_1151_MOESM3_ESM.pdf]

Description of Additional Supplementary Files

File Name: Supplementary Data 1
Description: Mass spectrometry analysis of CtIP TAP products.

File Name: Supplementary Data 2
Description: Mass spectrometry analysis of AUNIP TAP products.
